# Peptide-based versus standard polymeric enteral nutrition in the PICU: a multicenter observational study

**DOI:** 10.3389/fnut.2026.1824914

**Published:** 2026-07-15

**Authors:** Merve Misirlioglu Celik, Dincer Yildizdas, Faruk Ekinci, Sevinc Puren Yucel Karakaya, Muhterem Duyu, Ayse Asik, Abdullah Akkus, Fatih Akin, Hatice Feray Ari, Eylem Kiral, Ener Cagri Dinleyici, Hatice Elif Kinik Kaya, Nazik Yener, Mehmet Alakaya, Ali Ertug Arslankoylu, Busra Segmen, Ozden Ozgur Horoz, Hasan Ali Telefon, Tanil Kendirli, Eda Eyduran, Neslihan Zengin, Nihal Akcay, Ilyas Bingol, Sinan Yavuz, Ali Avci, Nazan Ulgen Tekerek, Murat Erdal, Feyza Incekoy Girgin, Makbule Nilufer Yalindag, Muhammed Udurgucu, Caglar Odek, Didar Arslan, Gurkan Atay, Hazal Ceren Tugrul, Hatice Albayrak

**Affiliations:** 1 Department of Pediatric Intensive Care Unit, Mersin University Faculty of Medicine, Mersin, Türkiye; 2 Department of Pediatric Intensive Care, Cukurova University Faculty of Medicine, Adana, Türkiye; 3 Department of Biostatistics, Cukurova University Faculty of Medicine, Adana, Türkiye; 4 Department of Pediatric Intensive Care, Medeniyet University Prof. Dr. Suleyman Yalcin City Hospital, Istanbul, Türkiye; 5 Department of Pediatric Intensive Care, Necmettin Erbakan University Faculty of Medicine, Konya, Türkiye; 6 Department of Pediatric Intensive Care, Aydin Adnan Menderes University Faculty of Medicine, Aydin, Türkiye; 7 Department of Pediatric Intensive Care, Eskisehir Osmangazi University Faculty of Medicine, Eskişehir, Türkiye; 8 Department of Pediatric Intensive Care, Ondokuz Mayis University Faculty of Medicine, Samsun, Türkiye; 9 Department of Pediatric Intensive Care, Adana Seyhan State Hospital, Adana, Türkiye; 10 Department of Pediatric Intensive Care, Ankara University Faculty of Medicine, Ankara, Türkiye; 11 Department of Pediatric Intensive Care, Manisa Celal Bayar University Faculty of Medicine, Manisa, Türkiye; 12 Department of Pediatric Intensive Care, Kanuni Sultan Suleyman Training and Research Hospital, Istanbul, Türkiye; 13 Department of Pediatric Intensive Care, Batman Training and Research Hospital, Batman, Türkiye; 14 Department of Pediatric Intensive Care, Akdeniz University Faculty of Medicine, Antalya, Türkiye; 15 Department of Pediatric Intensive Care, Marmara University, Pendik Training and Research Hospital, Istanbul, Türkiye; 16 Department of Pediatric Intensive Care, Marmara University Faculty of Medicine, Pendik Training and Research Hospital, Istanbul, Türkiye; 17 Department of Pediatric Intensive Care, Samsun Training and Research Hospital, Samsun, Türkiye; 18 Department of Pediatric Intensive Care, Bursa Uludag University Faculty of Medicine, Bursa, Türkiye; 19 Department of Pediatric Intensive Care, Ümraniye Training and Research Hospital, Istanbul, Türkiye; 20 Department of Pediatric Intensive Care, Sirnak State Hospital, Sirnak, Türkiye

**Keywords:** enteral nutrition, pediatric intensive care unit, peptide-based formula, standard polymeric formula, undernutrition

## Abstract

**Background:**

Nutritional guidelines recommend initiating enteral nutrition with standard polymeric formula (SPF) in critically ill children. Peptide-based formulas (PBF) have been increasingly used to improve enteral nutrition and reduce related complications. This study evaluated the clinical outcomes associated with PBF use compared with SPF in the PICU setting.

**Methods:**

This multicenter, prospective study included pediatric patients admitted to PICUs who received enteral nutrition. Patients receiving PBFs were compared with those receiving SPFs. To reduce baseline imbalances and potential confounding, propensity score matching with inverse probability of treatment weighting (IPTW) was applied.

**Results:**

A total of 574 patients were included. After IPTW adjustment, baseline characteristics were well balanced between groups. The rate of achieving target caloric intake by day 2 was significantly higher in the SPF group (*p* < 0.001); whereas no significant difference was observed by day 7 (mean difference = 0.7, *p* = 0.853). After adjustment, the incidence of nosocomial infections was significantly higher in the SPF group compared with the PBF group [23.5 vs. 17.6%; OR = 0.7 (95% CI, 0.5–0.9); *p* = 0.015]. Significantly greater reduction in proteins consumed on day 2 was observed in favor of the SPF group (mean difference −0.3, *p* < 0.001). Constipation was significantly more common in the SPF group (*p* = 0.010).

**Conclusions:**

Although early caloric and protein intake was higher in patients receiving standard polymeric formulas, outcomes were comparable by day 7. Lower infection rates and reduced need for mechanical ventilation were observed in patients receiving peptide-based formulas. Enteral formula selection should be individualized, and further studies are needed to clarify this subject.

## Introduction

Undernutrition is a pathological condition resulting from protein insufficiency, energy deficiency, or a combination of both. While malnutrition is a general term encompassing both undernutrition and overnutrition, this study specifically uses the term undernutrition to describe the state of nutrient deficiency in the study population, consistent with the clinical focus on energy and protein deficits. During the treatment of critically ill children in the pediatric intensive care unit (PICU), factors such as altered metabolic requirements and inadequate calorie intake increase the risk of developing undernutrition. All guidelines addressing the nutritional management of critically ill children recommend screening all admissions to assess nutritional status, with a particular emphasis on identifying those at high risk of undernutrition. Furthermore, these guidelines recommend the initiation of early enteral nutrition, provided there are no contraindications (1, 2).

The 2020 guidelines of the European Society of Pediatric and Neonatal Intensive Care (ESPNIC) on the nutritional management of critically ill pediatric patients recommend the initiation of enteral nutritional therapy using standard polymeric formulas (SPF) (2). However, a standardized management strategy for patients experiencing enteral nutrition intolerance has not yet been established. Enteral nutrition intolerance is common among pediatric patients. If nutritional requirements are not adequately met, the full benefits of enteral nutrition cannot be achieved, necessitating the management of adverse outcomes associated with undernutrition (1). The ESPNIC guidelines suggest considering peptide-based formulations (PBF) within the framework of “good medical practice” to improve tolerance and maintain enteral nutrition in pediatric patients who poorly tolerate or have contraindications to standard polymeric formulations (2, 3). In contrast, the 2017 guidelines of the American Society for Parenteral and Enteral Nutrition (ASPEN) on the nutritional management of critically ill patients do not provide specific recommendations regarding the choice of enteral formulas to be initiated (4).

To date, no clear consensus has been established regarding the optimal approach to managing enteral nutrition intolerance. The use of PBF represents one of the approaches to improve tolerance to enteral nutrition. Peptide-based formulations are enteral formulas containing proteins to have been enzymatically hydrolyzed into dipeptides and tripeptides. These hydrolyzed proteins are typically enteral nutrition products with a higher content of medium-chain triglycerides that are more easily absorbed and utilized (2–6).

The present study aims to evaluate the use of PBF in the PICU, examine their association with clinical outcomes, and provide a comprehensive comparison with SPF.

## Materials and methods

### Study populations and design

This prospective, observational, and multicenter study was conducted in 18 tertiary PICUs across Türkiye. The study included pediatric patients admitted to the participating PICU in the study centers who received hypercaloric enteral nutrition containing high levels of medium-chain fatty triglycerides (MCTs) and hydrolyzed protein (semi-elemental, PBF) throughout their stay in the PICU. Patients younger than 1 year or older than 18 years, those with an intensive care unit stay of less than 48 h or more than 6 months, and those with repeated admissions were excluded from the study. In cases of repeated admissions to the PICU during the study period, only the first admission was included in the analysis. Feeding practices were not standardized across centers; instead, formula choice was determined by local institutional protocols and the discretion of the treating physician. While patients in the PBF group were recruited from all 18 participating centers, data for the SBF group were provided by 2 of these centers which maintained comprehensive longitudinal records for both formula types.

This study is a prospective observational study. It was not registered in a public clinical trial database, as it did not involve any clinical interventions. The study was approved by the Çukurova University Non-Interventional Clinical Trials Ethics Committee (Decision No: 162/19, Date: January 9, 2026). The ethical approval was submitted to the participating centers, and each center obtained institutional approval from its respective hospital. The families of patients who met the study inclusion criteria were informed about the study, and parents who agreed to participate provided written informed consent.

### Data collection and definitions

A study form was distributed to all participating centers, and the PICU specialists responsible at each center completed the forms for data collection. Participating centers were required to record patients' demographic characteristics and clinical assessment data on the study forms. The variables collected included age, gender, diagnostic category, mode of nutrition, type of formula used for the initiation of enteral nutrition, formula-related complications, presence of feeding intolerance, required and delivered calorie intake, protein content, length of stay in the PICU, length of hospital stay, occurrence of nosocomial infections, need for respiratory support, and in-hospital mortality. Admission diagnoses were categorized according to organ systems.

Disease severity, mortality risk, and organ dysfunction scores, including the Pediatric Index of Mortality (PIM 2) (7) and Pediatric Risk of Mortality (PRISM III) (8), were assessed at the time of admission, whereas the Pediatric Logistic Organ Dysfunction (PELOD) score (9) was assessed during the follow-up period. The highest values were recorded. In the prospective study design, data from patients admitted to two designated PICU during the study period and received SPFs were recorded as the control group for comparison with the PBF group.

In this study, feeding intolerance (FI) was defined in accordance with the 2012 European Society of Intensive Care Medicine (ESICM) guidelines, characterized by the inability to reach the enteral nutrition target due to gastrointestinal symptoms or high gastric residual volumes (GRV). Specifically, FI was identified by the presence of vomiting/nausea, diarrhea (>2 mL/kg), abdominal distension, discomfort, or a GRV of ≥150 mL (or >3–5 mL/kg) (10). The GRV of ≥150 mL or >3–5 mL/kg is typically considered clinically significant. Furthermore, in bolus feeding, a GRV greater than half of the previous feeding amount, and in continuous feeding, a GRV exceeding the total feeding rate over a 2 h period, are considered clinically significant (5, 11).

### Assessment of nutritional status

To evaluate the nutritional status of critically ill pediatric patients in the present study, anthropometric measurements obtained at admission to the PICU and at discharge were analyzed. Height and weight measurements of the patients were obtained using calibrated devices and standardized techniques. A reference guide was distributed to all participating centers, and the collected data were subsequently used to calculate height-for-age, weight-for-age, and height-for-weight percentiles, as well as body mass index (BMI) *z*-scores, in order to assess the nutritional status of the patients. The z-scores of the anthropometric data were calculated using the CHILD METRICS software (12, 13). Patients were categorized according to their BMI z-scores as follows: morbid obesity (*z*-score > 3), obesity (*z*-score between 2 and 3, overweight (*z*-score between 1 and 2), normal (*z*-score between −2 and 1), underweight (*z*-score < −2), and severe underweight (*z*-score < −3).

The patients were evaluated for the mode of nutrition, the day of initiation of nutritional support, reasons for withholding enteral nutrition, the amounts of energy and protein received within the first 48 h and at the end of the 1st week, and whether the target calorie and protein requirements were achieved. The target caloric requirements of the patients were calculated using the Schofield equation (5, 14).

### Statistical analysis

The distribution of numerical variables was assessed using both visual and analytical methods to evaluate their adherence to a normal distribution. Continuous variables were summarized using descriptive statistics and reported as mean ± standard deviation (SD) for normally distributed data and as median with interquartile ranges for non-normally distributed or ordinal data. Comparisons between the two groups were performed using either the independent samples t-test or the Mann–Whitney U test, depending on the distribution of the data. Descriptive statistics for categorical variables included frequencies and percentages. Chi-square or Fisher's exact tests were used to compare nominal categorical data, whereas the Mann–Whitney U test was used to compare ordinal (ranked) variables between the two groups. A paired samples t-test was used to compare the changes in anthropometric measurements from the first day to the discharge day between the two groups (PBF vs. SPF).

Inverse probability of treatment weighting (IPTW) is one of the most popular approaches to account for confounding factors in observational studies. Choosing the IPTW methodology enhances the methodological quality of the study and reduces potential bias (15).

Demographic and clinical variables were summarized using frequencies and percentages for categorical data and as medians with interquartile ranges (IQRs) for continuous data. To account for baseline differences and reduce potential confounding, formula groups were balanced using propensity score methodology with IPTW (16).

Propensity scores, defined as the predicted probability of receiving peptide-based formula, were estimated using logistic regression models that included clinically relevant baseline covariates. IPTWs were then derived as follows: for patients in the standard formula group, the weight was 1/ps; for those in the PBF group, the weight was 1/ (1–ps), where ps denotes the estimated propensity score. Covariate balance between formula groups was assessed in both the unweighted (original) and weighted (pseudo) cohorts using standardized mean differences (SMDs). An SMD < 0.10 was considered negligible imbalance (17).

For binary outcomes, logistic regression models were fitted initially in an unadjusted (crude) form and subsequently weighted using stabilized IPTWs, with additional adjustment for covariates demonstrating minimal residual imbalance. Odds ratios (ORs) and corresponding 95% confidence intervals (CIs) were calculated for binary outcomes. For continuous outcomes, weighted linear regression models adjusted for residual confounders were employed. A two-sided *p*-value of less than 0.05 was considered statistically significant. All analyses were conducted using R software (version 4.1.1). Age, gender, severe undernutrition, comorbidities, PRISM score, nutritional status of the patient, and enteral nutrition program were evaluated, and IPTW was applied.

## Results

**Figure 1:**
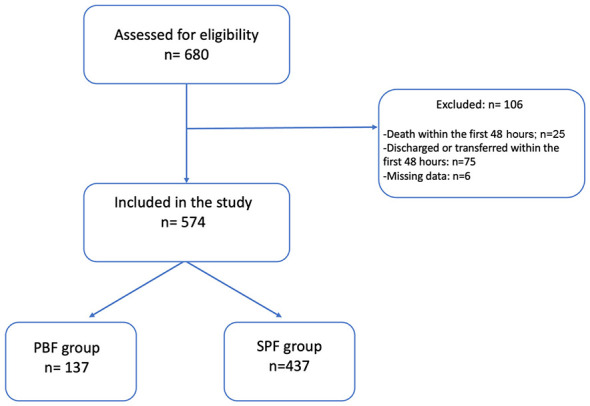
Flow diagram of the study population. PBF, protein-based formula; SPF, standard protein formula.

After excluding 106 patients (25 died within the first 48 h, 75 were discharged or transferred within the first 48 h, and 6 had missing data), 574 patients were included in the final analysis. Of these, 137 were in the PBF group and 437 were in the SPF group. The detailed patient recruitment and allocation process is illustrated in the flow diagram (Figure 1). Of the all patients, 54.9% (*n* = 315) were male, with a mean age of 68.77 ± 63.10 (min 1–max 214) months. The most common reason for admission to the intensive care unit was respiratory tract diseases (49.1%, *n* = 282). Comorbid conditions were present in 69.0% (*n* = 396) of all patients, with the most common comorbid condition being neuromuscular system disorders (55.8%, *n* = 221). The mean length of stay in the PICU was 22.12 ± 24.83 (min 3–max 242) days. Nosocomial infections were not observed in 78.9% (*n* = 453) of the all patients. The most common nosocomial infection was blood borne infections (47.9%, *n* = 58), followed by ventilator-associated pneumonia (42.1%, *n* = 51) and urinary tract infections (9.9%, *n* = 12). Mortality occurred in 62 patients (10.8%). During the study period, none of the observed mortalities were related to gastrointestinal events or complications of enteral nutrition. The observed mortalities were attributed to underlying critical conditions, primarily septic shock, multiple organ failure, and heart failure. Of all patients, 88.0% (*n* = 505) received mechanical ventilation support. Among these, 65.7% (*n* = 377) received invasive mechanical ventilation (IMV) and 47.2% (*n* = 271) received non-invasive mechanical ventilation [NIV or high-flow nasal cannula (HFNC)]; including 143 patients who received both modalities sequentially during their follow-up. Regarding nutritional support methods, 1.4% of patients received oral feeding, 83.1% *via* nasogastric/orogastric tube, and 15.5% *via* gastrostomy. Of the all patients, 80.1% (*n* = 460) received an intermittent enteral nutrition program. Constipation was significantly more common in the SPF group (*p* = 0.010). The demographic and nutritional characteristics of all patients in Tables 1, 2.

**Table 1:** Comparison of patients' demographic and nutritional characteristics between Peptide-based formulas and standard polymeric formulas.

	All patients (*n* = 574)	PBF (*n* = 137)	SPF (*n* = 437)	*p*-Value ^*^
Sex				
Female	259 (45.1%)	57 (41.6%)	202 (46.2%)	0.376
Male	315 (54.9%)	80 (58.4%)	235 (53.8%)	
Diagnosis at admission				
Respiratory diseases	282 (49.1%)	49 (35.8%)	233 (53.3%)	0.001
Neurological disorders	65 (11.3%)	14 (10.2%)	51 (11.7%)	
Infectious diseases	64 (11.1%)	18 (13.1%)	46 (10.5%)	
Postoperative care	50 (8.7%)	13 (9.5%)	37 (8.5%)	
Trauma	52 (9.1%)	27 (19.7%)	25 (5.7%)	
Cardiovascular diseases	32 (5.6%)	5 (3.6%)	27 (6.2%)	
Nephrological diseases	8 (1.4%)	1 (0.7%)	7 (1.6%)	
Hematological/oncological diseases	9 (1.6%)	4 (13.1%)	5 (1.1%)	
Gastrointestinal tract diseases	9 (1.6%)	6 (4.4%)	3 (0.7%)	
Intoxications	3 (0.5%)	0 (0.0%)	3 (0.7%)	
Presence of comorbid conditions	396 (69.0%)	92 (67.2%)	304 (69.6%)	0.598
Enteral nutrition method				
Peroral	8 (1.4%)	7 (5.1%)	1 (0.2%)	0.001
Nasogastric/orogastric tube	477 (83.1%)	110 (80.3%)	367 (84.0%)	
Gastrostomy	89 (15.5%)	20 (14.6%)	69 (15.8%)	
Enteral nutrition program				
Continuous	114 (19.9%)	52 (38.0%)	62 (14.2%)	< 0.001
Intermittent	460 (80.1%)	85 (62.0%)	375 (85.8%)	
Presence of nause/vomiting	95 (16.6%)	26 (19.0%)	69 (15.8%)	0.489
Presence of abdominal distention	136 (23.7%)	33 (24.1%)	103 (23.6%)	0.909
Presence of constipation	52 (9.1%)	5 (3.6%)	47 (10.8%)	0.010
Presence of diarrhea	74 (12.9%)	17 (12.4%)	57 (13.0%)	0.489
Development of metabolic abnormalities	60 (10.5%)	17 (12.4%)	43 (9.8%)	0.424
Nosocomial infections	121 (21.1%)	24 (17.5%)	97 (22.2%)	0.280
Mortality	62 (10.8%)	16 (11.7%)	46 (10.7%)	0.752
Mechanical ventilations	505 (88.0%)	117 (85.4%)	388 (88.8%)	0.294
According to body mass index on admission day				
Morbidly Obese (*z* score>3)	14 (2.4%)	1 (0.7%)	13 (3.0%)	0.233
Obese (*z* score 3–2)	33 (5.7%)	8 (5.8%)	25 (5.7%)	
Overweight (*z* score 2–1)	59 (10.3%)	13 (9.5%)	46 (10.5%)	
Normal (*z* score 1– −2)	270 (47.0%)	64 (46.7%)	206 (47.1%)	
Underweight (*z* score < -2)	52 (9.1%)	8 (5.8%)	44 (10.1%)	
Severely Underweight (*z* score < -3)	146 (25.4%)	43 (31.4%)	103 (23.6%)	
According to body mass index on discharge day				
Morbidly obese (*z* score>3)	16 (2.8%)	1 (0.7%)	15 (3.4%)	0.081
Obese (*z* score 3–2)	32 (5.6%)	7 (5.1%)	25 (5.7%)	
Overweight (*z* score 2–1)	61 (10.6%)	16 (11.7%)	45 (10.3%)	
Normal (*z* score 1– −2)	284 (49.5%)	65 (47.4%)	219 (50.1%)	
Underweight (*z* score < -2)	43 (7.5%)	5 (3.6%)	38 (8.7%)	
Severely underweight (z score < -3)	138 (24.0%)	43 (31.4%)	95 (21.8%)	
Reaching the target calorie day 2	356 (62.0%)	67 (48.9%)	289 (66.1%)	< 0.001
Reaching the target calorie day 7	371 (64.6%)	97 (76.4%)	274 (79.9%)	0.445

^*^Chi-square tests.

PBF, peptide-based formulas; SPF, standard polymeric formulas.

**Table 2:** Analysis of the numerical data for patients receiving peptide-based formulas and standard polymeric formulas.

	All patients (*n* = 574) Mean ±SD	PBF (*n* = 137) Mean ±SD	SPF (*n* = 437) Mean ±SD	*p*-value^*^
Age (months)	68.77 ± 63.10	95.55 ± 62.46	60.37 ±60.38	< 0.001
PIM 2 score	15 ± 13	13.13 ± 17.10	15.76 ± 23.01	0.152
PRISM III score	12.11 ± 10.27	13.25 ± 11.59	11.89 ± 10.64	0.203
PELOD II score	10.90 ± 9.09	12.62 ± 10.52	10.88 ± 10.28	0.080
IMV day	20.83 ± 27.19	22.57 ± 32.14	20.19 ± 25.17	0.452
NIV day	8.64 ± 12.82	10.78 ± 8.85	8.25 ± 13.38	0.246
Length of stay in the PICU (day)	22.12 ± 24.83	31.11 ±33.79	19.15 ± 20.24	< 0.001
Initiation of enteral feeding (day)	2.11 ± 1.52	2.48 ± 1.49	1.99 ± 1.50	0.001
EN calorie (day 2; *n* = 574; kcal/kg/day)	31.49 ± 35.47	21.09 ± 27.39	34.75 ± 37.07	< 0.001
EN calorie (day 7; *n* = 452; kcal/kg/day)	57.52 ± 40.04	50.94 ± 40.47	59.95 ± 39.66	0.030
EN protein (day 2; *n* = 574; gr/kg/day)	0.87 ± 1.01	0.59 ± 0.80	0.96 ± 1.05	< 0.001
EN protein (day 7; *n* = 452; gr/kg/day)	1.58 ± 1.14	1.40 ± 1.01	1.64 ± 1.18	0.045

^*^Independent parameters t tests.

PBF, peptide-based formulas; SPF, standard polymeric formulas; PICU, pediatric intensive care unit; PIM, pediatric index of mortality; PRISM, pediatric risk of mortality; PELOD, pediatric logistic organ dysfunction; IMV, invasive mechanical ventilation; NIV, non invasive mechanical ventilation; EN, enteral nutrition; kcal, kilocalories; kg, kilograms; gr, gram.

Regarding formula management, 13.8% (*n* = 79/574) of patients were initially given peptide-based formulas, while the remaining 86.2% (*n* = 495/574) received standard polymeric formulas. During the study period, 5.7% (*n* = 28/495) of the patients who started with SPF switched to PBF due to persistent FI symptoms and failure to achieve nutritional goals. Consequently, a total of 23.9% (*n* = 137/574) of patients received PBF at some point during their stay in the PICU. Clinical improvement following the initiation of PBF was defined as the regression of FI symptoms. Among the 28 patients who switched from SPF to PBF, gastrointestinal intolerance symptoms disappeared or significantly improved in 22 (78.6%) patients. In a study of 53 (38.7%, *n* = 53/137) patients who had nutritional intolerance before using PBF, 81.1% (*n* = 43/53) experienced a reduction in their symptoms after using PBF. At discharge from the PICU, 88.3% (*n* = 121/137) of the patients who received PBF continued on this formula, while 11.7% (*n* = 16/137) successfully returned to SPF after stabilization of their gastrointestinal function.

In this multicenter study, a total of 137 patients receiving PBF from 18 PICUs across Türkiye were evaluated over a 9-month period. In the PBF group, 58.4% (*n* = 80) of the patients were male, with a mean age of 95.55 ± 62.46 months. The most common reason for admission to the intensive care unit was respiratory tract diseases (35.8%, *n* = 49). Comorbid conditions were present in 67.2% (*n* = 92) of patients, with the most common comorbid condition being neuromuscular system disorders (51.5%, *n* = 47). The mean length of stay in the PICU was 31.11 ± 33.79 days. Nosocomial infections were not observed in 82.5% (*n* = 113) of the patients. The most common nosocomial infection was ventilator-associated pneumonia (54.2%, *n* = 13), followed by blood borne infections (37.5%, *n* = 9) and urinary tract infections (8.3%, *n* = 2). Mortality occurred in 16 patients (11.7%). Of the PBF patients, 85.4% (*n* = 117) received mechanical ventilation, 73.7% (*n* = 101) received invasive mechanical ventilation (IMV), and 29.9% (*n* = 41) received noninvasive mechanical ventilation [NIV or oxygen therapy *via* high-flow nasal cannula (HFNC)]. The patients were most commonly fed *via* a nasogastric or orogastric tube (80.3%, *n* = 110). Of the patients, 62% (*n* = 85) received an intermittent enteral nutrition program. The most common indications for the use of PBF were undernutrition or the risk of undernutrition (54.0%, *n* = 74), followed by intolerance to SPF (20.4%, *n* = 28), chylothorax (3.6%, *n* = 5), chronic diarrhea (2.9%, *n* = 4), short bowel syndrome (1.5%, *n* = 2), cystic fibrosis (1.5%, *n* = 2), and fat malabsorption (0.7%, *n* = 1). In 21 (15.3%) patients, no specific reason was given for initiating peptide feeding. Among the 28 patients who switched from SPF to PBF, gastrointestinal intolerance symptoms disappeared or significantly improved in 22 patients (78.6%). Before switching to or initiating PBF, 53 (38.7%) patients showed symptoms of feeding intolerance, while 84 (61.3%) did not. Following the switch to PBF, symptoms resolved in 43 of these 53 patients (81.1%), and significant clinical improvement was observed. Following PBF use, 113 (82.5%) patients did not experience symptoms of nutritional intolerance; however, 5 (3.6%) patients developed diarrhea, 5(3.6%) patients constipation, 3 (2.2%) patients nausea/vomiting, 4 (2.9%) patients increased gastric residual volume, and 7 (5.1%) patients abdominal distension. PBF was discontinued in only 5 (3.6%) patients.

The demographic and nutritional characteristics of patients in the two groups are presented in Table 1. There were no significant differences between the two groups regarding gender, presence of comorbidities, nausea and vomiting, abdominal distention, metabolic abnormalities, development of nosocomial infections, mortality, mechanical ventilation (IMV, NIV, and HFNC), nutritional status according to body mass index (admission/discharge), and achievement of the target caloric intake on day seven (*p* > 0.05). When examining the diagnoses on admission to the PICU, mode of nutrition, and nutrition programs, there were significant differences between the groups (*p* = 0.001, 0.001, and 0.000, respectively). Constipation was significantly more common in the SPF group (*p* = 0.010). Nutritional progress in patients was assessed at intervals based on whether or not they achieved their calorie targets. On day 2, 62% of the general population achieved their calorie targets (48.9% in the PBF group and 66.1% in the SPF group). By the end of day 7, target achievement had increased to 64.6% for the entire cohort (76.4% in the PBF group and 79.9% in the SPF group). By the time of discharge from the PICU, 80.8% of all patients had successfully achieved their prescribed calorie targets. The number of patients achieving the target caloric intake on day two was significantly higher in the SPF group (*p* < 0.001; Table 1). On Day 7, 472 patients remained in the PICU. Nutritional delivery was evaluated for the 452 patients (PBF group *n* = 124; SPF group *n* = 328) who were actively receiving enteral or parenteral support. The remaining 20 patients were temporarily not fed due to clinical reasons. The attrition from Day 2 (*n* = 574) was mainly attributed to discharge, with only 5 early deaths recorded, suggesting no significant attrition bias.

The comparison of continuous variables between patients receiving PBF and SPF is presented in Table 2. No significant differences were observed between the two groups regarding PICU scores and mechanical ventilation days (*p* > 0.05). Age, length of stay in the PICU, and time to the initiation of nutrition were significantly higher in the PBF group (*p* < 0.001, *p* < 0.001, and *p* = 0.001, respectively). Caloric intake of patients on days two and seven and protein intake on days two and seven were significantly higher in the SPF group (*p* < 0.001, *p* = 0.030, *p* < 0.001, *p* = 0.045; Table 2).

**Figure 2:**
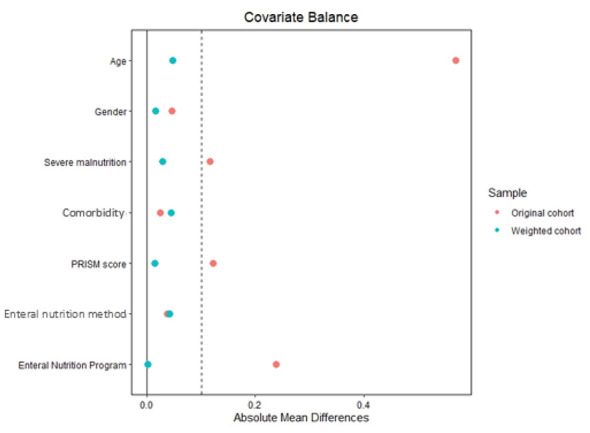
Absolute standardized mean differences before and after IPTW.

The present study evaluates the effects of PBF on clinical outcomes compared with SPFs. Baseline patient characteristics are presented in Table 3. Before IPTW, in addition to higher age and higher PRISM scores in the PBF group, severe undernutrition and continuous enteral nutrition program were also observed more frequently in this group. By contrast, the other variables listed in Table 3 appeared well balanced between the two formula groups in the original cohort. Following IPTW, the patient population was well balanced across groups for all characteristics (i.e., all SMDs were < 0.10; Figure 2).

**Table 3:** Patients' characteristics (before and after IPTW).

	Original cohort (before IPTW)			Balanced cohort (after IPTW)		
SPF (*n* = 437)	PBF (*n* = 137)	|SMD|	SPF	PBF	|SMD|
Age	32.0 (11.0–96.5)	93.0 (34.5–143.0)	0.570	44.0 (12.0–120.9)	53.7 (23.0–114.0)	0.048
Gender, *n* (%)						
Male	235 (53.8)	80 (58.4)	0.046	55.6	57.1	0.015
Female	202 (46.2)	57 (41.6)		44.4	42.9	
Severe undernutrition, *n* (%)						
No	360 (82.4)	97 (70.8)	0.116	78.4	75.6	0.028
Yes	77 (17.6)	40 (29.2)		21.6	24.4	
Comorbidity, *n* (%)						
No	133 (30.4)	45 (32.8)	0.024	30.4	25.9	0.044
Yes	304 (69.6)	92 (67.2)		69.6	74.1	
PRISM score	10.0 (5.0–14.5)	10.0 (5.0–18.0)	0.122	10.0 (5.0–15.0)	10.0 (5.0–15.0)	0.014
Enteral nutrition method, *n* (%)						
Nasogastric/orogastric	367 (84.0)	110 (80.3)	0.037	82.6	78.5	0.041
Gastrostomy	70 (16.0)	27 (19.7)		17.4	21.5	
Enteral nutrition program; *n* (%)						
Continuous	62 (14.2)	52 (38.0)	0.238	20.2	20.3	0.002
Intermittent	375 (85.8)	85 (62.0)		79.8	79.7	

Unless otherwise specified data was expressed as median (IQR).

|SMD|, absolute standardized mean difference.

PRISM, pediatric risk of mortality; PBF, peptide-based formulas; SPF, standard polymeric formulas.

In the weighted cohort, rates of metabolic abnormality were comparable between the SPF and PBF groups [10.6 vs. 8.1%; OR = 0.7 (95% CI, 0.5–1.1); *p* = 0.151], as were rates of intolerance [36.4 vs. 41.2%; OR = 1.2 (95% CI, 0.9–1.6); *p* = 0.099], rates of mortality [12.4 vs. 11.9%; OR = 0.9 (95% CI, 0.7–1.4); *p* = 0.800] and rates of reaching target caloric intake on day 7 [78.2 vs. 76.4%; OR = 0.9 (95% CI, 0.7–1.2); *p* = 0.524]. The rate of achieving the target caloric intake was significantly higher in the SPF group on day 2, but by day 7, it was comparable between the two groups (Table 4).

**Table 4:** Comparative effectiveness of SPF and PBF: categorical outcomes.

	Before IPTW				After IPTW			
	SPF *n* (%)	PBF *n* (%)	OR (95%CI)	*p* value	SPF	PBF	OR (95%CI)	*p* value
Metabolic abnormalities	43 (9.8)	17 (12.4)	1.3 (0.7–2.4)	0.485	10.6	8.1	0.7 (0.5–1.1)	0.151
Nosocomial infections	97 (22.2)	24 (17.5)	0.7 (0.5–1.2)	0.241	23.5	17.6	0.7 (0.5–0.9)	0.015
Enteral feeding intolerance	152 (34.8)	53 (38.7)	1.2 (0.8–1.8)	0.405	36.4	41.2	1.2 (0.9–1.6)	0.099
Mortality	46 (10.5)	16 (11.7)	1.1 (0.6–2.1)	0.825	12.4	11.9	0.9 (0.7–1.4)	0.800
Mechanical ventilations (IMV, NIV, and HFNC)	388 (88.8)	117 (85.4)	0.7 (0.4–1.3)	0.288	89.1	84.9	0.7 (0.5–0.9)	0.036
Reaching target calorie day 2	289 (66.1)	67 (48.9)	0.5 (0.3–0.7)	< 0.001	64.8	46.0	0.5 (0.4–0.6)	< 0.001
Reaching target calorie day 7	274 (79.9)	97 (76.4)	0.8 (0.5–1.3)	0.408	78.2	76.4	0.9 (0.7–1.2)	0.524

OR, odds ratio; aOR, adjusted odds ratio; CI, confidence interval.

IMV, invasive mechanical ventilation; NIV, noninvasive mechanical ventilations; HFNC, high flow nasal cannula oxygen; PBF, peptide-based formulas; SPF, standard polymeric formulas.

While unadjusted analyses showed no significant differences, IPTW-adjusted analyses revealed that infection and mechanical ventilation rates were significantly higher in the SPF compared with the PBF group [23.5 vs. 17.6%; OR = 0.7 (95% CI, 0.5–0.9); *p* = 0.015 and 89.1 vs. 84.9%; OR = 0.7 (95% CI, 0.5–0.9); *p* = 0.036, respectively; Table 4].

No significant difference was observed in the change in calories consumed on day 7 between the two groups (mean difference = 0.7, *p* = 0.853). However, a significantly greater reduction in calories consumed on day 2 was observed in favor of PBF group (mean difference −10.1, *p* < 0.001; Table 5). No significant difference was observed in the change in proteins consumed on day 7 between the two groups (mean difference = −0.1, *p* = 0.715). However, a significantly greater reduction in proteins consumed on day 2 was observed in favor of the SPF group (mean difference −0.3, *p* < 0.001; Table 5).

**Table 5:** Comparative effectiveness of SPF and PBF: continuous outcomes.

	Before IPTW				After IPTW	
	SPF	PBF	Difference^‡^	*p*	Difference^‡^	*p* ^*^
Length of stay in the PICU (day)	19.2	31.1	11.9	< 0.001	10.7	< 0.001
EN calorie day 2 (*n* = 574; kcal/kg/day)	35.0	21.5	−13.5	< 0.001	−10.1	< 0.001
EN calorie day 7 (*n* = 452; kcal/kg/day)	60.3	50.8	−9.5	0.018	0.7	0.853
EN protein day 2 (*n* = 574; gr/kg/day)	0.9	0.6	−0.3	< 0.001	−0.3	< 0.001
EN protein day 7 (*n* = 452; gr/kg/day)	1.6	1.4	−0.2	0.045	−0.1	0.715

^*^Lineer regression weighted by IPTW.

^‡^ Negative difference indicate a greater decrease in the PBF group, while positive difference indicate a smaller decrease compared with standard formula.

PICU, pediatric intensive care unit; EN, enteral nutrition; PBF, peptide-based formulas; SPF, standard polymeric formulas; kcal, kilocalories; kg, kilograms; gr, gram.

## Discussion

Isocaloric and polymeric enteral products should be the first-line products in critically ill pediatric patients in whom enteral nutrition is to be initiated. Hypercaloric polymeric products may be employed in cases where caloric requirements are increased and fluid restriction is required. When the proteins in an enteral product are hydrolyzed into peptides, the formula becomes a semi-hydrolyzed, oligomeric, or peptide-based enteral nutrition product. These products are typically rich in medium-chain triglycerides (MCTs) in terms of fatty acid composition. These semi-hydrolyzed peptide-based formulas exhibit more rapid digestion and absorption. Therefore, they are preferred in patients with digestive disorders or feeding intolerance (2–6).

In children with undernutrition, impaired gastrointestinal function leads to malabsorption and increased intestinal permeability. Appropriate nutritional intervention is the first step in the management of undernutrition. PBFs are associated with improved gastrointestinal tolerance and absorption, enhanced nitrogen balance, reduced diarrhea and bacterial translocation, improved fat absorption, and preserved as well as restored intestinal integrity comped with free amino acid-based or whole-protein formulas (18). In the present study, patients receiving standard polymeric formulas constituted the control group. Patients receiving hypercaloric peptide-based formulas containing high levels of MCT and hydrolyzed protein were analyzed.

Studies conducted in both pediatric and adult populations comparing formulas used in enteral nutrition have yielded various results (19–29). In their study examining postsurgical adult intensive care unit (ICU) patients, Sumritpradit et al. (19) analyzed the data of 9 patients receiving PBF and 10 patients receiving SPF. They found that patients receiving SPF experienced significant reductions in body weight, body mass index, and skeletal muscle mass. It was demonstrated that patients receiving PBF preserved their body weight and muscle weight and tended to achieve their caloric targets more rapidly compared with those receiving SPF (19). In the SPIRIT trial, a pilot-controlled study conducted to evaluate the effect of an enteral formulation designed to improve gastrointestinal tolerance in critically ill ICU patients, 46 patients receiving PBF and 44 patients receiving SPF were included. The study has shown that the use of PBF was effective in achieving daily protein targets; however, it proved ineffective in preventing the development of diarrhea or reducing the length of stay in the ICU (20). In a study involving medical, surgical, and cardiothoracic adult ICU patients, Seres et al. (21) reported that the use of PBF was associated with a reduction in feeding intolerance (21). In another study, Bhurayanontachai et al. (22) evaluated 63 critically ill adult patients and demonstrated that the use of PBF reduced feeding intolerance and proved effective in achieving target caloric intake (22).

In this multicenter cohort, which included a total of 574 critically ill pediatric patients, including 137 patients receiving PBF and 437 patients receiving SPF, no significant differences were observed between the two groups in terms of anthropometric parameters or feeding intolerance. However, constipation was more common in the SPF group. Calorie and protein intake on days 2 and 7 were significantly higher in the SPF group. The higher intake in the standard polymeric formula group was primarily a result of earlier initiation of feeding compared to the PBF group. Patients in the SPF group were more stable at an earlier stage, which may have allowed for a more increase in enteral volume. After IPTW adjustment, although there was no significant difference between the two groups in achieving target caloric intake on day 7, the rate remained significantly higher in the SPF group on day 2.

In our study, the lower rate of achievement of calorie targets on day 2 in the peptide-based formula group may be attributed to the baseline clinical condition of these patients and the later initiation of feeding. The time to initiation of enteral feeding was significantly longer in the PBF group, and there was a significant difference in PICU admission diagnoses even though there was no difference in baseline disease severity scores. This delay in initiation and progression of feeding leads to a lower percentage of patients achieving targets on day 2. In the study conducted by Aşik et al. (23), it was stated that the PBF group had higher protein intake and reached their calorie targets faster (23). Bhurayanontachai et al. (22) demonstrated in their studies that the use of peptide-based formulas enabled faster achievement of calorie targets in critically ill patients at high nutritional risk (22). While studies suggest that PBFs facilitate faster target achievement due to better tolerance, our findings may reflect the fact that PBFs are generally prescribed to patients who are more difficult to feed or who are in a more critical condition from baseline. Vidigal et al. (6) also noted in their study that PBFs are frequently initiated in patients with higher clinical severity (6).

Peptide-based formulas offer easier absorption and better tolerance. These formulas are often used selectively for patients with the most challenging nutritional challenges, and studies demonstrating their efficacy in other patient groups are weak. In our cohort, the SPF group performed better because there may be a more stable group of patients who were initially in a critical condition. While PBFs are designed to overcome nutritional challenges, their use in a more severely ill population may lead to slower results in achieving goals compared to a standard group with less baseline physiological stress.

In a study examining adult patients with acute gastrointestinal injury, including 71 patients receiving PBF and 121 patients receiving SPF, Wang et al. (24) reported no significant differences between the two groups in terms of mortality, length of ICU stays, or ventilator-free days. However, the study showed that patients receiving PBF achieved higher caloric and protein intake (24). In a study comparing the two formula groups in patients with acute pancreatitis, Tiengou et al. (25) reported that patients receiving PBF experienced less weight loss and had a shorter length of hospital stay (25). In contrast to these findings, the present study demonstrated that patients receiving SPF had a shorter length of hospital stay. This discrepancy may be attributed to the lower organ failure scores observed in patients receiving SPF, as well as the earlier initiation of enteral nutrition and the achievement of higher caloric and protein intake in this group.

In PICU practice, the use of peptide-based formulas is not limited to patients with overt gastrointestinal dysfunction or feeding intolerance. In our cohort, the primary indication for PBF is the presence of baseline malnutrition or at risk of malnutrition. In critically ill children, early achievement of protein and calorie targets is vital to prevent worsening of malnutrition. PBFs are often the preferred first-choice strategy in these vulnerable patients, regardless of gastrointestinal symptom status, to ensure optimal absorption and faster achievement of nutritional goals. PBFs are now utilized for a broader range of indications, including the early acute phase of critical illness and post-operative management of major surgeries, where impaired gut function is anticipated but not yet clinically evident (2, 4–6, 18–28). In a study examining 375 pediatric patients over a 3-year period, Ford and Gilbertson reported that PBF were well tolerated, and it was suggested that the indications for PBF may be broader than those currently specified (26). Similarly, this analysis demonstrated PBF was well tolerated, with only 3.6% of the patients requiring discontinuation due to intolerance. The main indications for the use of PBF in our study patients were undernutrition or the risk of undernutrition, followed by intolerance to SPF, chylothorax, chronic diarrhea, short bowel syndrome, cystic fibrosis, and fat malabsorption. In a retrospective study involving 132 children with cerebral palsy, Cai et al. (27) demonstrated that the use of PBF reduced malnutrition, improved gross motor function, and was associated with a low risk of adverse effects (27). Another study conducted in pediatric patients with cerebral palsy reported that the use of PBF improved anthropometric parameters, alleviated gastrointestinal intolerance symptoms, and normalized bowel movements (28).

Our literature review identified only a limited number of studies comparing PBF and SPF in critically ill children in PICU (6, 23, 29). Therefore, the authors of the present manuscript believe that the findings on the present prospective, multicenter study conducted in critically ill children will serve as a guide for future research in this field. Aşik et al. (23) published a single-center study in Türkiye comparing peptide-based formulas and fiber-enriched polymeric formulas (FEF) in terms of nutritional deficiencies, intolerances, and progress toward nutritional goals in a pediatric intensive care unit (PICU) population. The study included 225 critically ill children, 116 receiving PBF and 109 receiving FEF. Patients receiving peptide-based formulas showed earlier improvement in nutritional risk scores and faster achievement of calorie and protein goals (23). In this study, although patients receiving SPF had higher rates of reaching their target calorie intake and higher protein intake on day 2, there was no significant difference between the two groups on day 7. In a prospective study comparing the use of PBF and SPF in 291 patients in a PICU, Vidigal et al. (6) demonstrated that the length of PICU stay, duration of mechanical ventilation, and mortality rates were lower in the SPF group; however, the incidence of diarrhea was significantly lower in the PBF group (6). Consistent with their study, the present study found a shorter length of hospital stay in the SPF group. However, our study observed no significant differences between the groups in terms of duration of mechanical ventilation, mortality, or incidence of diarrhea. In our study; the higher age, longer stay, and later initiation of feeding in the peptide-based group are thought to be less about the formula itself and more likely to be a result of patients initially being clinically more severe and unstable, and physicians tending to prefer this formula for challenging cases.

In our study patients, the rate of nosocomial infections and the need for mechanical ventilation (IMV, NIV, and HFNC) were lower in the PBF group. In another study conducted in critically ill pediatric patients, Ibrahim et al. reported that enteral nutrition with PBF was better tolerated compared to SPF. Furthermore, a comparison between the SPF and PBF groups revealed no significant difference in terms of duration of mechanical ventilation, mortality rate, and length of stay in the PICU. However, the number of sepsis days were significantly lower in the PBF group. These findings were attributed to better weight gain and fewer feeding interruptions in the PBF group, leading to reduced bacterial translocation from the intestinal lumen into the systemic circulation, as well as to the potential immunomodulatory effects of the selected PBF (29). Similarly, our findings indicate this finding to improved gastrointestinal tolerance, which may reduce the systemic inflammatory response.

Despite IPTW adjustment, residual confounding by indication cannot be excluded, as PBFs were more frequently used in patients with higher disease severity and nutritional risk. Therefore, the observed lower infection and ventilation rates should be interpreted cautiously and not as causal effects.

In this multicenter cohort has some limitations. First, our study has a nonrandomized observational design, and the selection of the enteral formula was left to the discretion of the clinicians. This may have resulted in residual confounding due to unmeasured variables or factors not included in the model, despite comprehensive adjustment with IPTW. Second, the protocols regarding the timing of enteral nutrition initiation, the rate of feeding advancement, and the methods to calculate target caloric and protein requirements were not standardized across the participating centers. The heterogeneity in clinical practices across the study centers may have influenced nutritional outcomes, particularly in the early period. Third, the smaller number of patients receiving PBF compared with those receiving SPF limited the ability to perform certain subgroup analyzes. Other limitation of the study was that although IPTW adjustment was performed using available baseline demographic and clinical characteristics, some clinically relevant variables such as vasoactive support, sedation level, presence of shock or sepsis, ECMO use, and renal replacement therapy were not consistently available in this prospective multicenter dataset and therefore could not be included in the weighting model. These factors may influence both enteral feeding tolerance and clinical outcomes, and their absence may have resulted in residual confounding. In addition, although the PRISM score reflects overall illness severity, it may not fully capture specific therapeutic interventions and dynamic clinical conditions during PICU stay. The findings should therefore be interpreted with caution. One of the major limitations of the study was that the peptide-based formula groups were recruited from 18 centers, while the SPF control groups were derived from only two centers. This introduced a substantial risk of selection bias due to practical differences between the groups (i.e., variations in nutrition protocols and personnel expertise). The potential impact of this limitation must be taken into account when evaluating the study findings. Moreover, not stratifying the study group according to the underlying condition may limit the generalizability the study results. Due to its observational design, it is not possible to establish definitive causal relationships between formula types and clinical outcomes. The findings should be interpreted cautiously, as they represent clinical associations within a specific PICU setting. Future randomized controlled trials are needed to confirm these results.

## Conclusion

In the present study comparing peptide-based formulas and standard polymeric formulas for enteral nutrition in critically ill children, although patients receiving SPF had higher rates of achieving target caloric intake and higher protein intake on day 2, there was no significant difference between the two groups by day 7. Constipation was significantly more common in the SPF group. However, the incidence of nosocomial infections and the requirement for mechanical ventilation were lower in the PBF group. Although SPF enabled earlier caloric target achievement, this early advantage did not translate into superior clinical outcomes. The study concludes that the selection of enteral products should be customized according to the patient's clinical condition and tolerance. Further randomized controlled trials are needed to provide more robust evidence on this issue.

## Data Availability

The raw data supporting the conclusions of this article will be made available by the authors, without undue reservation.
